# Evolutionary Analyses Suggest a Function of MxB Immunity Proteins Beyond Lentivirus Restriction

**DOI:** 10.1371/journal.ppat.1005304

**Published:** 2015-12-10

**Authors:** Patrick S. Mitchell, Janet M. Young, Michael Emerman, Harmit S. Malik

**Affiliations:** 1 Molecular and Cellular Biology Graduate Program, University of Washington, Seattle, United States of America; 2 Division of Basic Sciences, Fred Hutchinson Cancer Research Center, Seattle, United States of America; 3 Division of Human Biology, Fred Hutchinson Cancer Research Center, Seattle, United States of America; 4 Howard Hughes Medical Institute, Fred Hutchinson Cancer Research Center, Seattle, United States of America; University of Michigan, UNITED STATES

## Abstract

Viruses impose diverse and dynamic challenges on host defenses. Diversifying selection of codons and gene copy number variation are two hallmarks of genetic innovation in antiviral genes engaged in host-virus genetic conflicts. The myxovirus resistance (*Mx*) genes encode interferon-inducible GTPases that constitute a major arm of the cell-autonomous defense against viral infection. Unlike the broad antiviral activity of MxA, primate MxB was recently shown to specifically inhibit lentiviruses including HIV-1. We carried out detailed evolutionary analyses to investigate whether genetic conflict with lentiviruses has shaped *MxB* evolution in primates. We found strong evidence for diversifying selection in the MxB N-terminal tail, which contains molecular determinants of MxB anti-lentivirus specificity. However, we found no overlap between previously-mapped residues that dictate lentiviral restriction and those that have evolved under diversifying selection. Instead, our findings are consistent with MxB having a long-standing and important role in the interferon response to viral infection against a broader range of pathogens than is currently appreciated. Despite its critical role in host innate immunity, we also uncovered multiple functional losses of *MxB* during mammalian evolution, either by pseudogenization or by gene conversion from *MxA* genes. Thus, although the majority of mammalian genomes encode two *Mx* genes, this apparent stasis masks the dramatic effects that recombination and diversifying selection have played in shaping the evolutionary history of *Mx* genes. Discrepancies between our study and previous publications highlight the need to account for recombination in analyses of positive selection, as well as the importance of using sequence datasets with appropriate depth of divergence. Our study also illustrates that evolutionary analyses of antiviral gene families are critical towards understanding molecular principles that govern host-virus interactions and species-specific susceptibility to viral infection.

## Author Summary

Evolutionary analyses have the potential to reveal not only biochemical details about host-virus arms-races but also the nature of the pathogens that drove them. Primate MxB was recently shown to restrict the replication of primate lentiviruses, including HIV-1. However, we find that positive selection in primate *MxB* is incongruent with known molecular determinants of lentiviral restriction. This suggests that MxB has antiviral activity against a broader range of viruses than is currently appreciated. We also identified multiple losses of *MxB* in mammals, as well as rampant recombination between *Mx* paralogs, which has distorted gene orthology. Our study illustrates the importance of evolution-guided functional analyses of antiviral gene families.

## Introduction

Ancient, pathogenic viruses have played a major role in shaping the extant host innate immune repertoire. Understanding how pathogen-driven evolution has shaped host-virus interfaces can reveal insights into the molecular basis of cross-species transmission, including human susceptibility to zoonoses [1]. The genetic signature of diversifying (positive) selection distinguishes many host antiviral genes, indicating their involvement in a long-standing genetic conflict with viral pathogens. Such genetic conflict has also driven gene copy number expansion in several mammalian antiviral genes, allowing further diversification of pathogen defense. For instance, the *TRIM5* antiviral gene is present in one copy in primates but has expanded to 6–7 copies in mice and other mammals [2,3]. Similarly, primates encode seven members of the *APOBEC3* antiviral gene family whereas mouse genomes encode only one [4,5].

Unlike the *TRIM5*, *APOBEC3* or other antiviral gene families, the copy number of myxovirus resistance (*Mx)* gene appears to be relatively static, with two copies in both primate and mouse genomes. Mx proteins are interferon-inducible dynamin-like large GTPases. They are composed of a highly conserved GTPase domain (GD), which is connected to a helical stalk by a hinge-like bundle-signaling element (BSE) [6]. Previous work has shown that human MxA and both murine Mx1 and Mx2 proteins have broad and potent activity against a diverse range of RNA and DNA viruses [7,8]. In contrast, the antiviral activity of human MxB appears to be much more narrow, only recently having been shown to restrict HIV-1 and other primate lentiviruses [9–12].

In this study we employed a detailed evolutionary approach to address the basis for the apparent stasis of *Mx* gene copy number and the discrepancy in antiviral breadth of Mx homologs. Using maximum-likelihood approaches, we found strong evidence of diversifying selection in the N-terminal region of primate *MxB* genes in contrast to the previously observed diversifying selection in loop L4 of *MxA* [13]. Surprisingly, signatures of *MxB* diversifying selection do not overlap with previously-mapped lentiviral-restriction determinants. We therefore conclude that simian lentiviruses have not driven the rapid evolution of primate *MxB*. Our analysis instead suggests that MxB plays a central and conserved role in the interferon response to a broader range of pathogens than is currently appreciated. Extending our analysis to other mammalian *Mx* genes, we find that multiple, lineage-specific exchanges have occurred between *Mx* paralogs throughout mammalian evolution. These gene conversion events have led to both the preservation of key enzymatic and structural features of Mx GTPases, as well as the acquisition of new antiviral specificity via the complete conversion of *MxB*-like genes to a *MxA*-like state. Our findings highlight the impact of diversifying selection and gene conversion on the functional repertoires of antiviral gene families.

## Results

### Did lentiviruses drive the evolution of primate *MxB*?

We wished to investigate whether the primate *MxB* gene has been subject to pathogen-driven diversifying selection. A previous analysis reported that the primate *MxB* gene had not evolved under diversifying selection, even though it did find evidence for positive selection for some individual sites (see below) [14]. In contrast, our previous analysis of primate *MxA* found strong evidence of positive selection at both the gene and codon level [13]. Although this variance could reflect genuine differences in selective pressures that have acted on the two paralogs, we also considered the possibility that lower sampling of *MxB* sequences in the previous report may have led to reduced power to detect selection [15].

**Fig 1:**
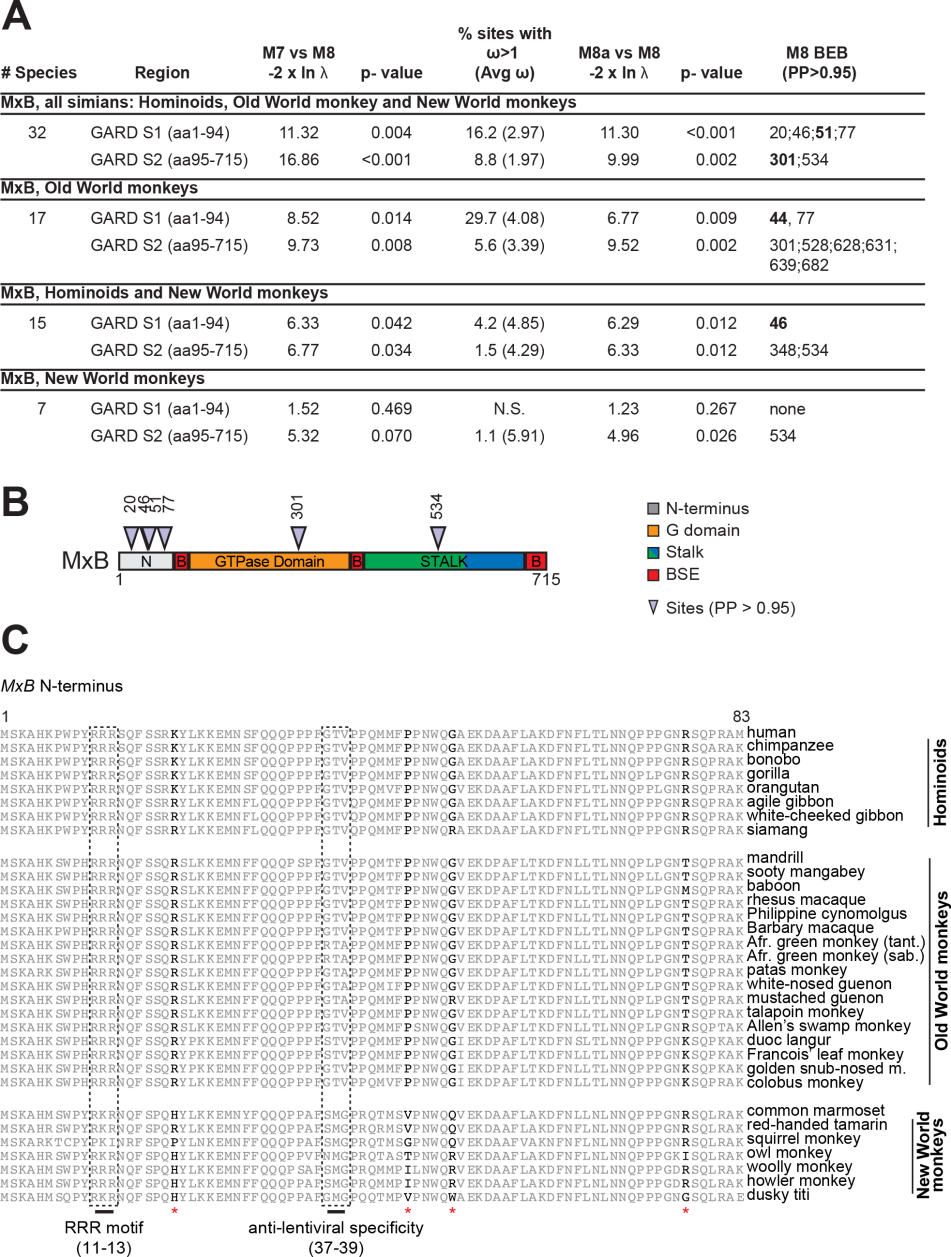
Rapid evolution in *MxB* is concentrated in the disordered N-terminus. **(A)** Results of positive selection analyses of primate *MxB*. Because GARD analysis suggests that the N-terminal 94 amino acids of primate MxB could have a different evolutionary history from the rest of the gene, we analyzed selective pressures separately on each segment ("GARD S1" and "GARD S2"). The "M8 BEB" column lists rapidly evolving sites with dN/dS > 1 (posterior probability (PP) ≥ 0.95), as determined by Bayes Empirical Bayes (BEB) implemented in PAML's model M8, with coordinates relative to human MxB. Sites that were also identified using REL (Bayes Factor > 50) are bolded. N.S., Non Significant. See also S1 Fig and S3 Table. (**B)** MxB (715 amino acids) is depicted highlighting rapidly evolving sites identified in our "all-simian" analysis (triangles) and major features: GTPase domain (orange), bundle-signaling element (“B,” red), MxA- and MxB-specific amino-termini (“N,” grey) stalk domain (colors indicate prior nomenclature of middle (green) and GTPase effector domain (blue), respectively). Note that the last 14 codons were not included in the analysis due to sequence ambiguity based on the primer design. **(C)** A protein alignment of the primate MxB N-terminus (amino acids 1–83) is shown for 32 primate species. Positively selected residues identified in the "all-simian" analysis are marked by a red asterisk. Experimentally determined regions important for MxB anti-lentivirus activity [14,21] are highlighted in dashed boxes. Please refer to S1 and S2 Datasets.

We, therefore, cloned and sequenced *MxB* from 21 hominoid, Old World monkey and New World monkey species for a total of 32 *MxB* sequences after including sequences from public databases. Maximum likelihood tests were implemented using the PAML [16] and HyPhy [17] suite of programs to detect whether rates of non-synonymous changes (dN) exceeded synonymous changes (dS) (dN/dS > 1 implies positive selection). Recombination can yield false signatures of positive selection [18]. We used a genetic algorithm for recombination detection (GARD) [19,20] to show that *Mx* genes did indeed undergo recombination during their evolutionary history. We therefore carried out selection analyses only on *MxB* gene segments for which evolutionary history was determined to be uniform by GARD (Fig 1A). For all analyzed GARD segments, we found strong evidence for positive selection in primate *MxB* (M7 vs. M8, *P* < 0.001) (Fig 1A). Our findings support a role for *MxB* in a long-standing and recurrent host-virus conflict during primate evolution.

Our analyses also revealed six codons in primate *MxB* that showed strong evidence of diversifying selection (model M8, Bayes Empirical Bayes (BEB) posterior probability (PP)>0.95) (Fig 1A and 1B). Four of these six rapidly evolving residues are located in the disordered N-terminus of MxB (amino acids 1–83) (Fig 1B). The MxB N-terminus determines both its localization to the nuclear pore as well as its antiviral specificity against simian lentiviruses [14,21–24]. The positive selection in MxB contrasts with the positive selection in MxA, which is concentrated in the loop L4 (S1B Fig) [13]. Although our previous analysis also found evidence of positive selection in the MxA N-terminus, we identified no sites in the loop L4 of MxB in primates that had a high posterior probability of having evolved under diversifying selection (Fig 1, S1A Fig). Our findings appear to be at odds with a recent analysis of *MxB* evolution in mammals, which concluded that positive selection is centered on the MxB L4 [25]. However, this previous analysis was based on a broad range of mammals and did not account for the possibility of *MxA-MxB* recombination, which might have confounded the analysis (see below). Despite the high similarity of MxA and MxB GTPases [26], our finding that diversifying selection is centered on distinct surfaces (L4 versus N-terminus), together with their divergent cellular localization (MxA is cytoplasmic whereas MxB is nuclear) [22,27,28], suggests that different pathogens have uniquely shaped MxA and MxB antiviral surfaces during primate evolution.

The N-terminus of MxB has been shown to be essential for its antiviral activity against lentiviruses [14,21–24,29]. We considered whether simian lentiviruses could be responsible for driving positive selection in primate *MxB*. If so, we would expect that the amino acids that govern anti-lentiviral specificity of MxB would also be the amino acids that are under diversifying selection as has been previously observed for APOBEC3G and TRIM5 [30–33]. We therefore compared the residues that evolved under diversifying selection with two regions in the MxB N-terminus previously identified as molecular determinants of MxB anti-lentivirus activity (Fig 1C), a triple-arginine motif (RRR11-13), and residues 37–39. To our surprise, we found no overlap between either of these molecular determinants and positively selected sites. Mutation of the RRR11-13 motif abolishes MxB anti-lentiviral activity [21]. Moreover, anti-HIV-1 activity can be conferred on the highly diverged canine MxB by restoring the RRR11-13 motif [21]. We find that the triple arginine motif arose in the common ancestor of simian and prosimian primates and has since been strictly conserved, except for in New World monkeys in which it has degenerated multiple times (Fig 1C). Residues 37–39 are known to dictate MxB's differential activity against different lentiviruses. Specifically, the group O HIV-1 chimeric CMO2.5 strain is sensitive to human but not African green monkey (AGM) MxB. Similarly, the HIV-1 P207S capsid mutant is sensitive to rhesus macaque but not AGM MxB. Functional differences between AGM and rhesus macaque MxB map to N-terminal residues 37–39, especially residue 37 [14]. We find no evidence that recurrent diversifying selection has acted on either the RRR motif or residues 37–39 (Fig 1). Formally, target recognition of the lentiviral capsid may span a broader region of the MxB N-terminus although alanine-scanning mutagenesis of these sites did not appear to disrupt anti-HIV-1 activity [21]. Given that Old World monkeys are the primary primate lineage infected by lentiviruses, we also restricted our analysis to Old World monkey sequences; analysis of only these species might therefore be expected to better reveal lentiviral-driven selection. Again, we found positive selection in *MxB* but not in residues shown to confer lentiviral specificity (Fig 1). Likewise, if we exclude Old World monkeys and analyze only hominoids and New World monkeys, we still observe positive selection (Fig 1). Analysis of New World monkeys alone is also weakly suggestive of positive selection in the GARD 2 segment (Fig 1), but our analysis (only 7 NWM species) lacks the statistical power for confident interpretation of this result; a denser sampling of this clade is needed. Thus, despite the occurrence of intense diversifying selection in the MxB N-terminus, our results strongly imply that the only known antiviral activity of MxB, i.e., towards primate lentiviruses, cannot explain the rapid evolution of primate *MxB*.

### Comparison of positive selection analysis to previously published datasets

Our analysis of positive selection in *MxB* is discrepant with two previous findings, which reported that (A) *MxB* has not evolved under positive selection in primates [14], and that (B) the sites under positive selection in mammalian *MxB* are predominately located in loop L4 [25]. Here, we explore the sources of these discrepancies, which may help inform future analyses of antiviral genes.

Busnadiego et al. [14] performed three NSsites tests to detect positive selection in primate *MxB*, one of which was significant. However, the single significant analysis (model 0 vs. 3) tests variability in the ω ratio among sites and does not constitute a test of positive selection [34]. In contrast, both analyses that are designed to detect positive selection (i.e., model 1 vs. 2, or 7 vs. 8) were not significant. However, it should be noted that Busnadiego et al. based their analysis on 12 *MxB* sequences, of which 3 were macaque species and 5 were hominoids, making this dataset relatively shallow. Previous work has demonstrated that species representation strongly influences the robustness and inference of positive selection analyses [15]. In addition, although ten sites were reported to be under positive selection by both REL (posterior probability > 0.9) and M3 (probability > 0.99); (the former is a valid test of positive selection), false positives may arise from the analysis of shallow datasets [35]. Thus, the difference in our ability to detect positive selection in primate *MxB* is the result of the increased number and diversity of *MxB* sequences in our analysis wherein we included 32 primate species dispersed throughout the phylogenetic tree.

Sironi et al. [25] also identified positive selection in *MxB* based on an analysis of 29 eutherian mammals. However, while our analysis identifies the MxB N-terminus as the “hotspot” of diversifying selection, they identified the MxB L4 (Fig 1). It is formally possible that primates have experienced a unique evolutionary history relative to other eutherian mammal lineages, which might explain the incongruence between our results. However, the conclusion that MxB L4 has been a target of selection in mammals should be tempered for two reasons: i) Large divergences, such as those found across 29 mammals, are prone to dS saturation leading to the underestimation of dS (i.e., false positives) [34]. ii) The failure to account for recombination can also lead to false positives [18]. Recombination was not detected in their dataset despite our finding of recurrent recombination in a similarly sampled analysis (see below). Although we do not know the exact set of sequences assayed in Sironi et al., we note that 7 of 31 species listed in their S1 Table encode pseudogenized or gene converted copies of *MxB*. Our analyses highlight the merit of dense sampling within mammalian orders compared to broad sampling across orders, which is better suited to identify conserved features [36].

### Dynamism of *Mx* genes in eutherian mammals

**Fig 2:**
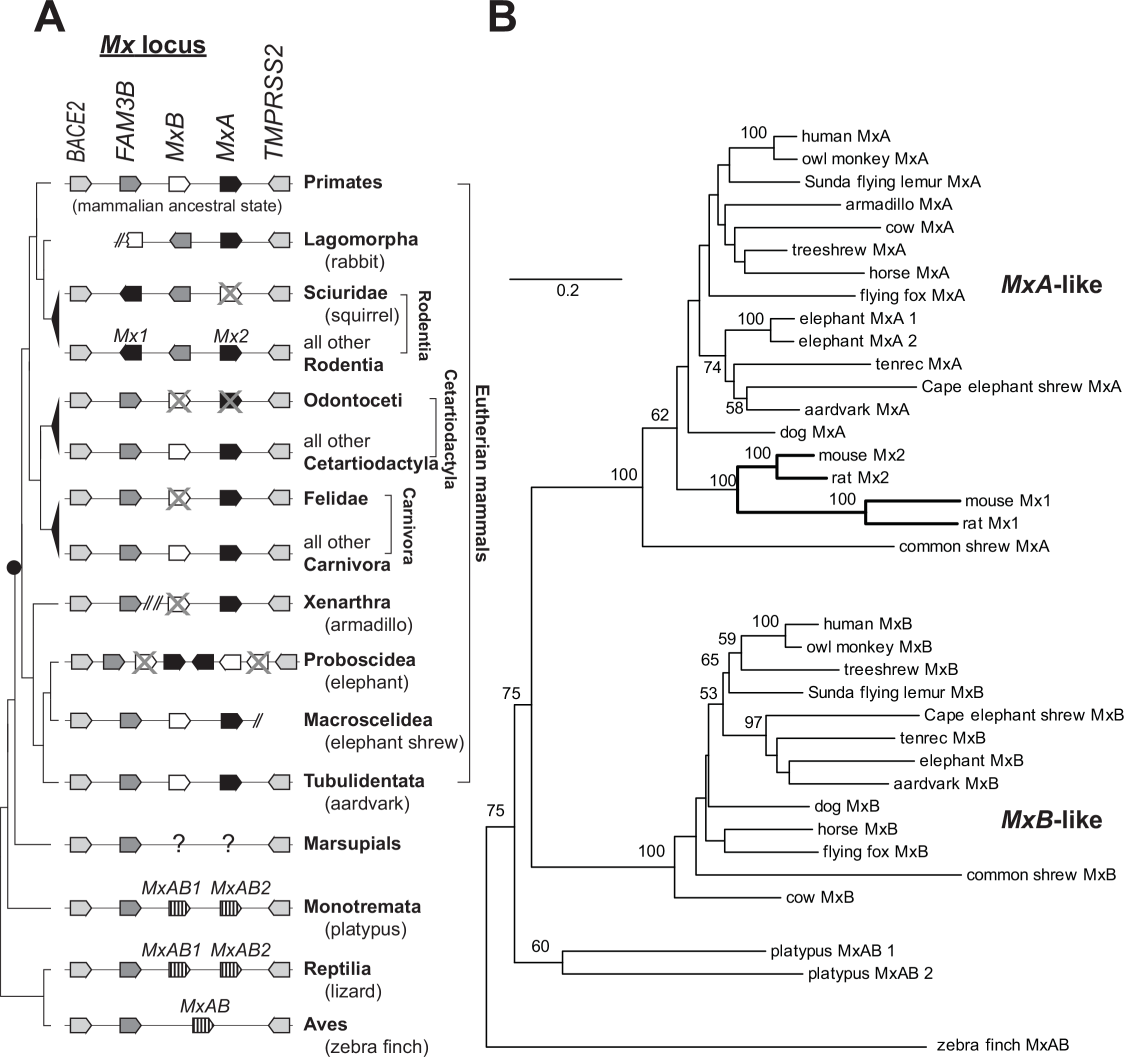
The complement and phylogenetic relationship of *Mx* genes in eutherian mammals. **(A)** The orientation and relative position of genes in the *Mx* locus from representative mammalian orders and selected outgroups (marsupials, platypus, Reptilia and Aves) are represented by black (*MxB*-like), white (*MxA*-like), hatched (*Mx* ancestral), dark gray (FAM3B) or light gray (BACE2/TMPRSS2) pentagons. Slashes indicate instances in which the *Mx* locus is present on distinct genomic scaffolds. Cases in which inferences are based on one representative species are indicated by parentheses. The inferred ancestral *Mx* locus is present in species from the orders Carnivora, Cetartiodactyla, Primate and Tubulidentata (aardvark), with instances of *Mx* gain and loss (grey “X” represent pseudogenes, "?" symbols represent complete loss) indicated. See also S2 Fig. Altered gene order and orientation in lagomorphs and rodents suggests locus reorganization. The cartoon phylogeny (left) shows the species tree [40], with the inferred origin of the eutherian mammal *Mx* duplication shown as a black circle. **(B)** A phylogeny of *Mx* genes from representative mammals and selected outgroups reveals that *MxA*-like and *MxB*-like genes form two major clades. Bootstrap support for nodes greater than 50 are shown. Rodent *Mx* paralogs are indicated with bold branches to highlight that rodents have two *MxA*-like genes, rather than a single gene in each clade. Please refer to S3 Dataset.

Many antiviral gene families have undergone dynamic, lineage-specific changes in copy number, presumably as a mechanism for gaining new antiviral specificities without losing existing functions [37]. In contrast to other antiviral gene families, previous studies have found that *Mx* gene copy number is relatively static [25,38]. However, this apparent stasis may be misleading. For instance, a recent report found that *Mx* genes have been lost in Odontoceti cetaceans (toothed whales) [39]. To more comprehensively determine the evolutionary dynamics of *Mx* genes in mammals, we performed phylogenetic analyses of mammalian *Mx* paralogs from at least one representative of all sequenced mammalian orders. We found shared synteny of the *Mx* locus throughout terrestrial vertebrates (Fig 2A). Both human and mouse genomes encode two *Mx* genes, but as previously described [38], rodents have lost the *MxB*-like gene and instead encode two *MxA* orthologs (*Mx1* and *Mx2*). Platypus genomes encode two *Mx* genes that both appear ancestral to the eutherian *MxA* and *MxB* lineages; we term them here *MxAB1* and *MxAB2* (Fig 2). We were unable to identify any *Mx* genes in any of three marsupial genome sequences (tammar wallaby, opossum, Tasmanian devil) and suggest that *Mx* gene(s) were lost from the common marsupial ancestor. We therefore infer that *MxA* and *MxB* genes diverged at or just prior to the origin of the eutherian mammal lineage.

Within eutherian mammals, our phylogenetic analyses revealed surprisingly poor resolution; many nodes have less than 50% bootstrap support and some discordance at well-supported nodes of the mammalian phylogeny (Fig 2B) [40]. These discrepancies are not entirely unexpected for rapidly evolving antiviral genes, and likely reflect complex evolutionary histories of *Mx* genes in multiple mammalian lineages (see below). Nevertheless, we were able to conclude that most eutherian mammalian genomes encode both *MxA*-like (orthologous to human *MxA*) and *MxB*-like (orthologous to human *MxB*) genes. Further, elephants encode two closely related intact *MxA* genes in addition to *MxB*; this *MxA* duplication appears to have occurred since divergence from the sister order Macroscelidea (*e*.*g*., elephant shrew) (Fig 2). With the exception of the loss of both *MxA* and *MxB* genes in toothed whales [39], we found evidence for an intact MxA gene in all surveyed eutherian mammal species. In contrast, we found that *MxB* has been lost at least three additional, independent times in Rodentia, Felidae and Xenarthra (Fig 2 and S2 Fig). Therefore, *MxB* loss has been tolerated on multiple occasions during eutherian mammal evolution despite the fact that its importance as an antiviral factor has been experimentally demonstrated.

Phylogenetic and synteny analyses allowed us to propose a hypothetical scenario for the loss of the *MxB* gene in mouse. We found that the ancestral configuration of the mammalian *Mx* locus was *Bace2(+);Fam3B(+);MxB(+);MxA(+);Tmprss2(-)* (Fig 2A). In the rabbit genome (Lagomorpha, an outgroup to Rodentia), an inversion occurred in the *Mx* locus such that *MxB* and *Fam3B* have opposite orientations relative to the ancestral locus (Fig 2A). Distinct rearrangements appear to have taken place in Rodentia, represented by squirrel and mouse genomes, leading to a *Bace2(+);Mx1(-);Fam3B(-);Mx2(+);Tmprss2(-)* configuration (Fig 2A). Intriguingly, squirrel *Mx2* is an *MxB*-derived pseudogene, whereas mouse *Mx2* is an *MxA*-derived intact gene (S2 Fig). Based on this, we propose that mouse *Mx2* originated as a result of complete gene conversion by *Mx1* (*MxA-like)* (Fig 2A), possibly preceded by loss of the ancestral *MxB*-like gene in rodents.

### Recurrent gene conversion between *Mx* genes preserves enzymatic and structural elements but scrambles phylogenetic relationships

**Fig 3:**
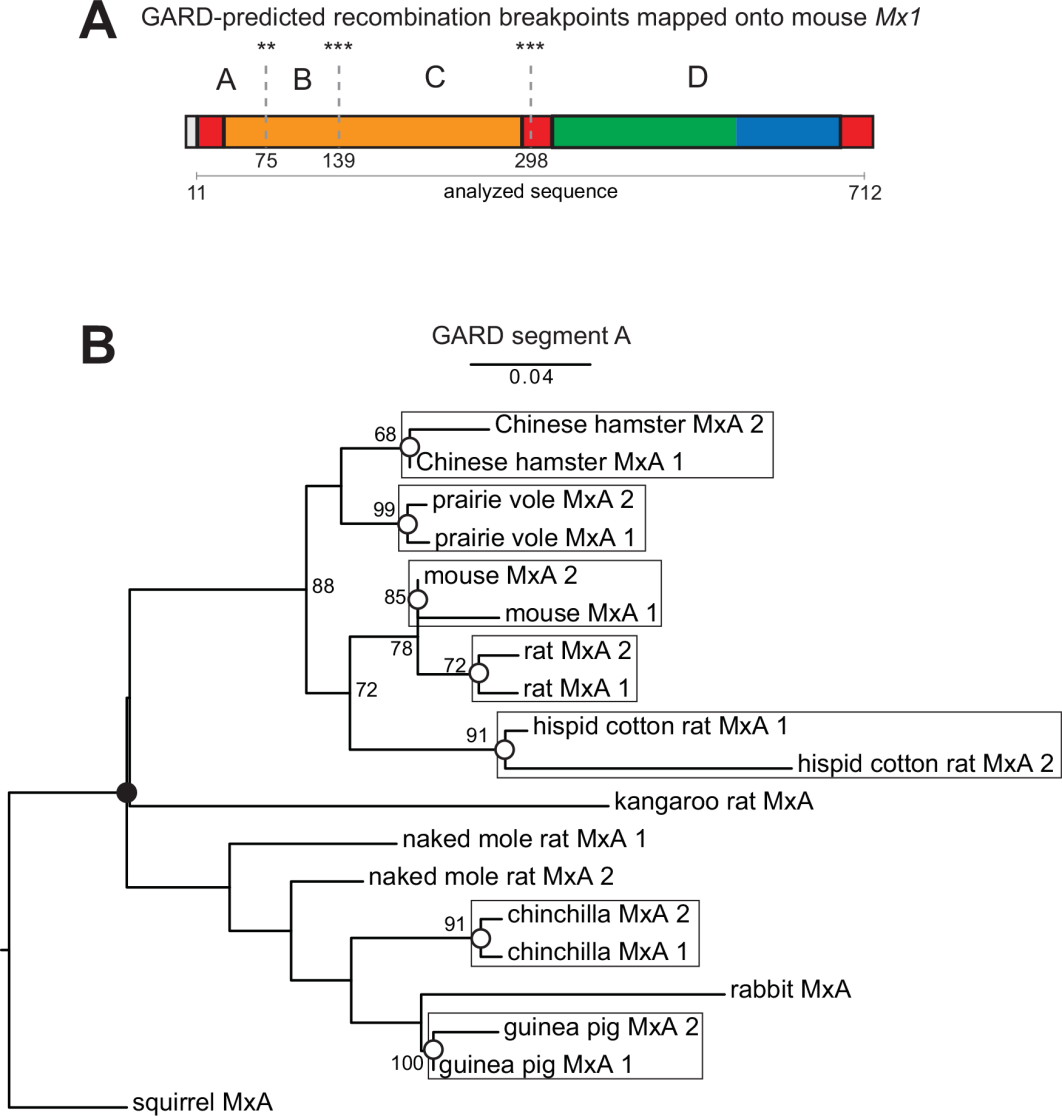
Recurrent gene conversion of *Mx* genes scrambles phylogenetic relationships in rodents and closely related species. **(A)** GARD analysis suggests that recurrent recombination events between rodent and rabbit *Mx* paralogs have resulted in at least four segments of the gene with distinct evolutionary histories (evidence ratio >100). Supported recombination breakpoints (***, *P* < 0.01; **, *P* < 0.05) and the location of GARD segments are shown on a linear schematic of the mouse Mx1 protein, colored as in Fig 1B (also see S3 Fig). **(B)** A maximum-likelihood phylogeny of selected rodent and rabbit *Mx* genes was generated from an alignment of the *Mx* GARD segment A using PHYML. Shared ancestral (black circle) or more recent lineage-specific (white circles) recombination events leading to *Mx* paralog homogenization are highlighted. Boxes highlight instances in which paralogous *Mx* genes cluster more closely with each other than with orthologs. Please refer to S4 Dataset.

We further investigated gene conversion of rodent *Mx* genes using GARD [19,20] to identify putative recombination breakpoints in a multiple sequence alignment. We found that recurrent gene conversion has occurred between *Mx* genes throughout rodent evolution such that various *Mx* gene segments have distinct, incongruous evolutionary histories (Fig 3A and S3 Fig). We used PHYML to generate bootstrapped phylogenies of each segment identified by GARD; this analysis confirmed that different segments have different phylogenies, with key nodes indicative of recombination supported by strong bootstrap values. For example, a phylogeny based on GARD segment A indicates that for the majority of rodent species the *Mx1* and *Mx2* genes are more closely related to each other than to the orthologous gene in related species (Fig 3B, white circles). We also use mVISTA [41] to determine that gene conversion tracts can extend into intronic sequences (S3 Fig). The phylogenetic grouping of mouse-like rodents also suggests that more ancestral conversion events have occurred. We estimate that at least eight independent gene conversion events have occurred in this region alone between *Mx1* and *Mx2* genes in the eight surveyed rodent species (indicated at specific nodes in Fig 3B). However, given that our sampling is limited to sequenced genomes, this is likely an underestimate of the degree of gene conversion/recombination during rodent *Mx* evolution (S3 Fig). Thus, a high frequency of gene conversion events has scrambled the phylogenetic relationships between rodent *Mx* genes.

**Fig 4:**
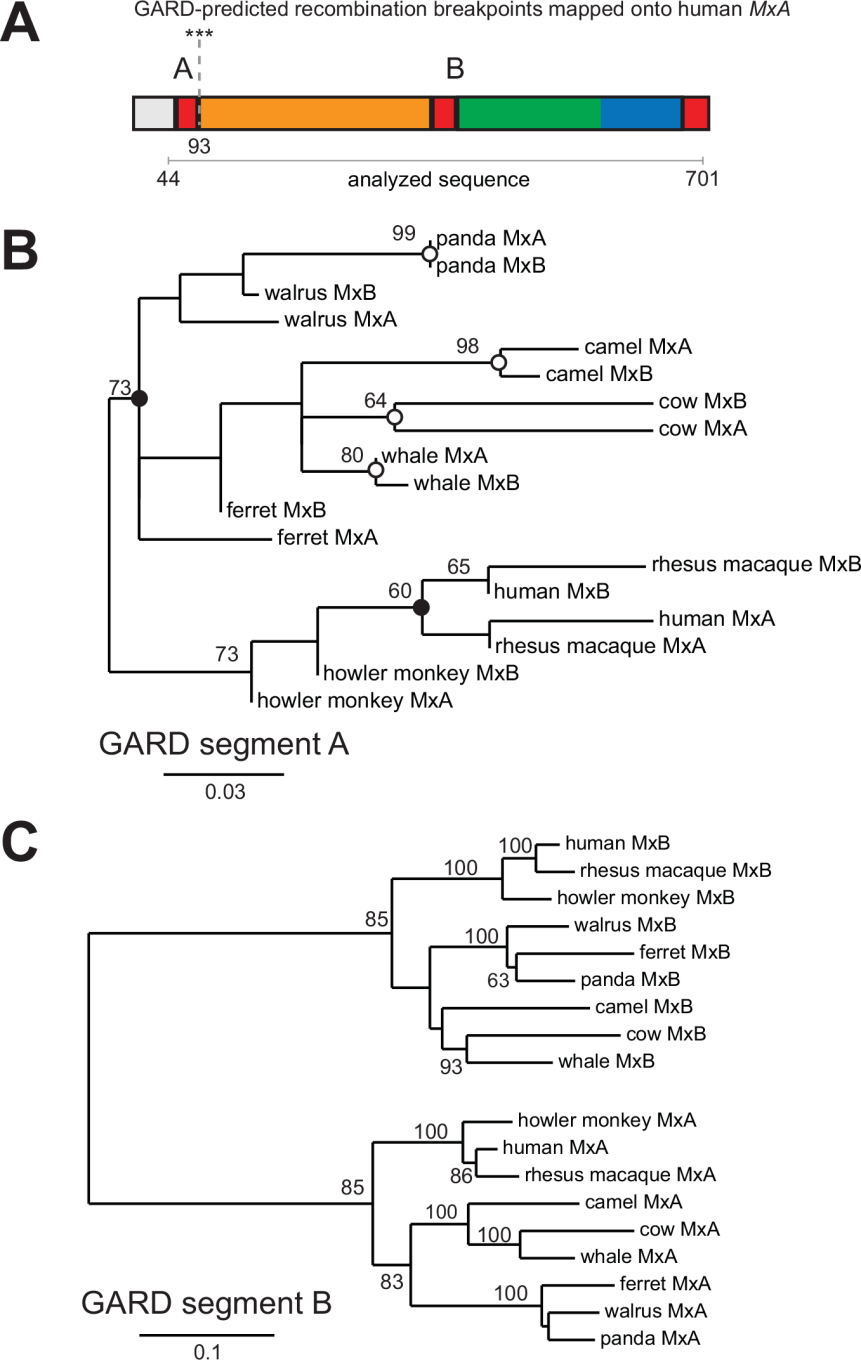
Recombination substantially impacts the BSE and GTPase domain of *MxA* and *MxB* via gene conversion. **(A)** GARD analysis suggests that recurrent gene conversion/recombination events between *Mx* paralogs have resulted in at least two segments of the gene with distinct evolutionary histories (evidence ratio >100). Supported recombination breakpoints (***, *P* < 0.01; **, *P* < 0.05) and the location of GARD segments are shown on a linear schematic of the human MxA protein, colored as in Fig 1B (also see S4 Fig). (**B, C)** We used PHYML to generate maximum-likelihood phylogenies of the two GARD-defined segments of *MxA* and *MxB* from selected primate, carnivore and ungulate species. Gene conversion that homogenized GARD segment A in the shared ancestor of each mammalian order (black circles) or in specific lineages (white circles) is indicated in panel B (also see S4 Fig). Note that a full-length *MxA-MxB* phylogeny is similar to that of GARD segment B. Please refer to S4 Dataset.

We next extended our survey of possible gene conversion between *Mx* genes beyond rodents to other mammalian genomes. We found at least two *Mx* gene segments have distinct evolutionary histories among eutherian mammalian *Mx* paralogs (Fig 4). In contrast to the nearly complete gene conversion/recombination between rodent *Mx* paralogs (Fig 3 and S3 Fig), in other mammals recombination is more localized (S4 Fig). For instance, the regions that coincide with the second coding exon of human *MxA* and *MxB* are remarkably similar to each other (S4 Fig). These exons correspond to the Mx bundle-signaling-element BSE α1B (amino acids 84–116 in human MxA) and GTPase domain α1G (amino acids 117–147) (S1 and S4 Figs). α1G contains the highly conserved P-loop (G1), which is an essential GTP-binding element that interacts with the α- and β-phosphates of bound nucleotide [42].

Similarly, we also found that within primate, carnivore and ungulate mammals, the BSE-GTPase domain-encoding segments (GARD segment A, Fig 4) from *MxA* and *MxB* cluster with each other, instead of by species; independent phylogenetic analyses support GARD's finding of recombination with high bootstrap support. Based on these observations, we conclude that there has been recurrent gene conversion/recombination between the BSE-GTPase domain-encoding exons of *MxA* and *MxB* genes that occurred early after the separation of the different mammalian orders (Fig 4B, black circles). As in rodents, there have been additional recent exchanges in some mammalian lineages (Fig 4B, white circles). We note that this frequent intergenic recombination is the likely cause of the apparent poor resolution of the *Mx* gene phylogeny in mammals (Fig 2B). Therefore, gene conversion of the BSE-GTPase domains has contributed to the genetic exchange of critical functional elements between the two *Mx* genes over different evolutionary timeframes.

## Discussion

In our study, we have found that despite the apparent stasis of *Mx* gene copy number, both rapid evolution and recurrent gene conversion have led to a highly diversified complement of *Mx* genes in mammals. We find that *MxB*, like *MxA*, has evolved under positive selection, which argues that it has had a broader role in antiviral defense than is currently appreciated. *MxB* has been lost in some lineages, whereas in others, it has been converted into an *MxA*-like gene. Moreover, recurrent recombination has exchanged important enzymatic and structural motifs between *MxA* and *MxB*. Thus, both pathogenic pressure and recombination between paralogs has shaped the extant specificity of Mx antiviral proteins in different extant mammalian genomes. Discrepancies between our findings and previous studies of *MxB* evolution [14,25] provide a practical illustration of several important considerations when performing evolutionary studies: (a) it is necessary to use sufficiently dense phylogenetic sampling to have the power to detect positive selection; (b) recombination can confound evolutionary history and must be accounted for in studies of selective pressures; and (c) use of species sets that are too diverged can cause problems due to difficulties in accurately estimating synonymous rates.

### What drives *MxB* evolution in primates?

Molecular arms races between simian lentiviruses and their infected primate hosts have been ongoing for at least 5 million years [31,32]. Evidence for lentivirus-driven evolution has been identified in other restriction factors [32] indicating that ancient, simian lentiviruses have imposed a dominant selective pressure on primate antiviral genes. Despite the coincidence of rapid evolution being centered on the MxB N-terminal tail, we find no evidence of diversifying selection having acted on either the RRR11-13 lentiviral-restrictive motif or residue 37, the two known molecular determinants of MxB anti-lentivirus activity. Previously, comprehensive triple-alanine-scanning mutagenesis of MxB’s N-terminal domain (amino acids 1–91) effectively ruled out any other determinants of antiviral activity against HIV-1 [21]. We therefore fail to find evidence that primate lentiviruses have driven diversifying selection in primate *MxB*. However, since only a subset of primate lentiviruses have been directly tested for MxB restriction, it is possible that some of the diversifying selection we have mapped might correspond to either primate lentiviruses that have not been tested, or to ancient lentiviruses that no longer exist.

On the other hand, since MxB anti-lentivirus activity does not require GTP binding or hydrolysis [9,10], if lentiviruses were the driving target of primate MxB we might have expected MxB GTPase motifs to have degenerated. Contrary to this expectation, we find that all GTP binding motifs are strictly conserved in all surveyed mammals that encode an intact *MxB* gene (S1C Fig). Preservation of the MxB GTPase domain may either imply a house-keeping function as previously suggested [43], or an antiviral requirement for GTPase activity against other (non-lentiviral) pathogens, as is seen with *MxA* [44,45]. The recurrent loss of MxB in various mammalian lineages suggests that an important housekeeping role for MxB is either unlikely or would have to be highly lineage-specific.

Despite the ability of MxB to inhibit primate lentiviruses, its evolution is inconsistent with a recurrent “arms race” scenario with that group of pathogens. The absence of overlap in *MxB* diversifying selection with previously-mapped lentiviral restriction determinants contrasts with other restriction factors such as TRIM5α and TRIMcyp, in which genetic innovation directly correlates with capsid-binding and antiviral restriction [33,46–50]. It is possible that the selective pressure exerted on lentiviruses by MxB *in vivo* may be weak compared to other restriction factors, thereby reducing the likelihood or intensity of an MxB-capsid arms race. In single round or spreading infections, MxB mediates 5–20 fold restriction [9,10], lower than the >100-fold restriction demonstrated for APOBEC3G, TRIM5α, and TETHERIN [12,51–53]. Another possibility is that the site of MxB restriction in capsid does not allow for viral escape. This possibility seems unlikely since MxB-resistant capsid mutants can be readily selected *in vitro* [14,54]. On the other hand, though MxB-escape mutant viruses might arise readily, it is possible that they are rendered more susceptible to interaction with other capsid-binding host restriction factors (e.g., TRIM5alpha, TRIMcyp). If this is true, MxB may play a crucial albeit indirect role in constraining capsid evolution as part of a multifaceted interferon response. Finally, it is formally possible that the molecular determinants of MxB anti-lentivirus activity do not mediate direct binding to lentiviral capsid but instead mediate its interaction with a cellular cofactor, leading to purifying rather than diversifying selection.

Our findings are reminiscent of previous studies in which we found diversifying selection of the primate *Bst-2/Tetherin* restriction factor at nef- but not vpu-interacting sites [55]. While such analyses cannot irrefutably prove that nef drove diversifying selection of primate *Tetherin*, it strongly argues that vpu did not. Using a similar rationale, we conclude that a different lineage(s) of viruses, distinct from currently known simian lentiviruses, have shaped *MxB* evolution in primates. We considered whether LINE-1 retrotransposons may have driven *MxB* evolution based on the recent discovery that they are also restricted by MxB [56]. However, LINE-1 restriction is independent of MxB's N-terminal tail [56], so it is unlikely that a conflict with retroelements drove diversifying selection in the N-terminus of primate MxB. Instead, we hypothesize that the evolutionary signatures of diversifying selection in MxB N-terminus, together with the evolutionary constraint acting on its GTPase domain, implicates its action against a widespread family (or families) of as-yet-identified pathogens. Interestingly, the signature of diversifying selection distinguishes the mode of MxA and MxB antiviral specificity. Just as positive selection in MxA is centered in the loop L4, which mediates its target recognition [13], we predict that individual changes in the MxB disordered N-terminus may also define MxB antiviral specificity.

### Recurrent gene conversion has shaped Mx antiviral repertoires in mammals

MxA (cytoplasmically localized) and MxB (nuclear pore localized) proteins restrict different classes of viruses [38], which likely explains their stable retention as paralogs in most mammals. In light of this fact, it is surprising that gene conversion has resulted in the loss of *MxB* in rodents, followed by retention of an additional *MxA*-like gene in some. Interestingly, mouse *Mx1* and *Mx2* appear to have subfunctionalized, diverging in their antiviral range by localizing to different cellular compartments (cytoplasmic and nuclear) [22,27,28]. Therefore, the conversion of *MxB* to *MxA* in some rodents allowed for the refinement of MxA-like antiviral activity against cytoplasmic and nuclear replicating viruses, even at the expense of ancestral MxB-like activity. Although mouse Mx1 is nuclear, its localization is distinct from MxB (human MxB localizes to the nuclear pore whereas mouse Mx1 forms discrete nuclear foci). Furthermore, the evolutionary acquisition of a C-terminal NLS on Mx1 is unique to mouse-like rodents [57]. Unlike rodents, however, pseudogenization of *MxB* in other lineages (e.g. armadillo, felids and squirrel) does not appear to be have been driven by *MxA* diversification. Instead, these may reflect cases in which MxB-targeted pathogens went extinct, relaxing selective pressure to maintain MxB activity. A similar loss of constraint may also explain the dual loss of both *MxA* and *MxB* in toothed whales [39].

Rodents appear to be distinct from other mammals in their *Mx* gene conversion profiles. In other eutherian mammals, including primates, gene conversion between *MxA* and *MxB* appears to be largely restricted to a region encoding parts of the BSE and GTPase domains. Such localized gene conversion could be the result of an uncharacterized recombination hotspot. However, it is also possible that this is the indirect result of selective constraint. For example, a swap of the BSE-GTPase domain is least likely to deleteriously impact the antiviral repertoire of the Mx paralogs. As a result, gene conversions spanning the BSE-GTPase domain, but not other domains, might be more easily tolerated. In contrast to this ‘tolerated conversion’ model, it is also possible that the frequent gene conversion of the BSE-GTPase domain is instead directly favored by selection. We speculate that gene conversion may serve as an adaptive mechanism (akin to diversifying selection) to escape antiviral antagonism. For instance, if the MxB BSE-GTPase domain were the direct target of viral antagonism, replacing it with the diverged but functionally equivalent MxA BSE-GTPase domain might be a rapid means to escape antagonism without compromising function. In support of this latter idea, there are residues under diversifying selection in the GTPase domain of Mx proteins in primates [13], which might be the result of a genetic conflict to escape viral antagonists.

Many antiviral gene families have undergone extensive, lineage-specific copy number variation, reflecting distinct bouts of pathogen-driven evolution and highlighting genetic variation in the antiviral repertoires of even closely related species. At first glance, *Mx* antiviral genes appear to belie the highly dynamic nature of antiviral gene copy number variation. Instead, closer examination reveals the highly dynamic evolution of *Mx* antiviral genes, with both diversifying selection having driven changes in presumed viral specificity domains and recombination homogenizing the catalytic domains of Mx proteins. Due to both these evolutionary forces, there is little likelihood of *Mx* ‘orthologs’ maintaining functionally analogous antiviral repertoires. At the very least, our analysis raises the need for caution in functional assignments of *Mx* genes based on the established “Mx1" and "Mx2” nomenclature, especially for rodents. Indeed, similar whole or partial gene conversion events are likely to have shaped many mammalian antiviral multigene families in which paralogs are present in genetic proximity to each other [2,4,58].

## Materials and Methods

### Cloning and sequencing

Primate fibroblasts were purchased from the Coriell Cell Repository. Cells were cultured in DMEM (Gibco) supplemented with 10% FBS and 5% penicillin/streptomycin. RNA was isolated from cultured primate fibroblasts using the RNeasy kit (Qiagen). RT-PCR to obtain primate *MxB* cDNA sequences was performed using HiFi one-step RT-PCR (Invitrogen) with the following primers:

Hominoid

F 5’—ATGTCTAAGGCCCACAAGCCTTGG—3’

R 5’—GTGGATCTCTTTGCTGGAGAATTGACAGAGTG—3’

Old World monkey

F 5’—ATGTCTAAGGCCCACAAGTCTTGGC—3’

R 5’—RTGGATCTCTTCGCTGGAGAATTGACAGAG—3’

New World monkey

F 5’—ATGTCTAAGGCCCACAKGTCTTGGCC—3’

R 5’—CTCTGTAAATTCTCCAGTGAAGRGATCCACGACTACAAAGACGACGACAAATGA—3’

Gel extracted products were directly sequenced (Sanger method). Contigs were assembled using CodonCode Aligner. Detailed information on primate species and cell lines used in this study can be found in S2 Table. Sequences have been deposited in GenBank (accession numbers KT698228-KT698252).

### Sequence collection, alignment and phylogenetic analyses

*Mx* genes were retrieved from publically available databases (non-redundant nucleotide collection, reference genomic sequences, high-throughput genomic sequences and whole-genome shotgun contigs) using BLASTN or TBLASTN from at least one representative of all sequenced mammalian orders (S1 Table). After including sequences obtained through RT-PCR, multiple sequence alignments were conducted using CLUSTALW or MUSCLE, and adjusted manually. To determine the evolutionary relatedness of *Mx* genes, maximum-likelihood phylogenies (1000 replicates) were constructed using PhyML under the GTR substitution model either locally or through the phylogeny.fr website [59,60]. Analysis of *Mx* locus synteny was evaluated in Ensembl using the Comparative Genomics Alignment tool for standard reference genomes or by retrieving genomic sequences and using BLASTN analyses to determine order of *Mx* homologs and flanking genes.

### Detection of recombination

We examined alignments for evidence of recombination using the GARD algorithm [20] from the HyPhy package [17], run on local computers. Briefly, for each in-frame alignment to be analyzed, we first used HyPhy's NucModelCompare to suggest the best-fitting nucleotide substitution model (using as input a maximum-likelihood tree generated from the entire alignment using PhyML [59] and the GTR model). We then supplied the alignment and best-fitting substitution model to GARD, using the general discrete model of site-to-site rate variation with 3 rate classes. Breakpoints assigned by GARD were independently assessed by generating bootstrapped (n = 1000) maximum-likelihood phylogenies in PhyML.

### Positive selection analysis

Maximum-likelihood tests were performed with CODEML implemented with the PAML software suite [16]. Input trees were generated from each alignment using PHYML [59] with the GTR+I+G nucleotide substitution model; using alignment-specific trees is more appropriate than using the species tree in this case, given that recombination has scrambled the orthologous relationships of these genes. Mx coding sequence alignments were fit to NS sites models that disallow (M7 or M8A) or allow (M8) ω > 1. Models were compared using a chi-squared test (degrees of freedom = 2) on twice the difference of likelihood values to derive *P* values reported in Fig 1A and S1A Fig). Analyses were robust to varying codon frequency models (F3x4 and F61); results from analyses using model F3x4, which we empirically find to be more conservative, are shown in all figures. The model 7 vs 8 comparison was also robust to the initial omega value used (0.4, 1 or 1.5); model 8a cannot be run with other initial omega values. In cases where a significant difference (*P* < 0.01) between M7 versus M8 (or M8A versus M8) was detected, the Bayes Empirical Bayes (BEB) analysis was used to identify codons with ω > 1 (reporting values with posterior probability ≥ 0.95). The depiction of positively selected sites on crystal structure representations was carried out in PyMol (pymol.org).

We also used the REL algorithm of the HyPhy package to detect selected sites [17]—we uploaded each alignment to the DataMonkey website, used the model selection tool to select the most likely evolutionary model, and used that model and the alignment as input to the REL algorithm.

## Supporting Information

S1 Table Identifiers for sequences used in this study. Genbank accession numbers for all Mx gene sequences used for the analyses in this study.
(DOCX)

S2 Table Primate species and cell lines used in the study. We report the source cell lines from which the MxA and/or MxB sequences were obtained for this study. In all cases, *MxA* and *MxB* gene sequences were derived from the same sample. Primate *MxB* sequences cloned in this study are indicated (Genbank Accession numbers KT698228-KT698252). WNPRC, Washington National Primate Research Center. TNPRC, Tulane National Primate Research Center.
(DOCX)

S3 Table Sites with dN/dS > 1 as detected by REL (GARD segments analyzed individually). Please see Methods for details about how the analyses were carried out and residues putatively evolving under diversifying selection identified.
(DOCX)

S1 Fig Distinct signatures of positive selection in primate *MxA* and *MxB*, and conservation of the *MxB* GTPase domain. **(A)** and **(B)** Positive selection analyses of primate *MxA* and *MxB*. To directly compare the pattern of positive selection between primate *MxA* and *MxB*, we reanalyzed both genes using an equivalent dataset of 25 primate species. We performed analysis only on segments of each gene that show uniform evolutionary history as assessed by GARD. Each row shows results from one segment of the gene. The "M8 BEB" column lists rapidly evolving sites with ω > 1 (posterior probability (PP) ≥ 0.95), as determined by Bayes Empirical Bayes (BEB) implemented in PAML's model M8. Sites that were also identified using REL (Bayes Factor > 50) are bolded. N.S., Non Significant. The location of rapidly evolving sites is indicated using triangles on a linear schematic of each gene as described in Fig 1B. We also show these sites on the crystal structure representations of (A) MxB (PDB 4WHJ) [26] and (B) MxA (PDB 3SZR) [61] proteins. We confirmed our previous findings [13] that positively selected residues are clustered in MxA L4 (5/8 rapidly evolving codons); variation at these L4 sites shifts the antiviral specificity of human MxA against orthomyxoviruses [13]. In contrast, we found that the primate MxB L4 is highly conserved whereas its N-terminal tail is rapidly evolving. These two distinct “hotspots” of diversifying selection are consistent with the differential antiviral specificities of MxA and MxB. Thus, our analyses suggest that like the L4 of MxA [13], the MxB N-terminal tail defines its antiviral specificity through direct interaction with viral target(s). **(C)** A protein alignment (left) of MxB GTP binding motifs from the same representative species in Fig 2B. The position of each motif (based on human MxB) is indicated below the alignment, with key residues highly conserved in all Dynamin-like GTPases [62] highlighted in green. A top view of the MxB crystal structure (PDB 4WHJ) [26] is shown (right) with coloring as in Fig 1B. The highly homogenized region of the BSE and GTPase domain encoded by *MxB* exon 3 is in pink. Nomenclature of alpha helices is based on human MxA [61].
(EPS)

S2 Fig Losses of *MxB* in mammalian evolution. In addition to rodents, we found three independent *MxB* loss events in *Felidae*, armadillo, and squirrel, all of which encode a single *MxA* gene (Fig 2). In each case, we used the dotter algorithm [63] to sensitively compare the genomic region containing the human *Mx* locus with *Mx* loci from the corresponding genome assemblies (*Felis catus*, domestic Abyssinian cat, felCat5 assembly; *Panthera tigris*, Siberian tiger, PanTig1.0 assembly; *Dasypus novemcinctus*, nine-banded armadillo, dasNov3 assembly; *Spermophilus tridecemlineatus*, squirrel, speTri2 assembly). Dotter comparisons incorporating human *Mx* gene annotations allowed us to identify residual patches of *MxB* pseudogene homology in cat, tiger, armadillo and squirrel. In each panel, we show an alignment of part of *MxB* from representative species. For each species, we show nucleotide sequence above and a translation below, using the reading frame relative to that sequence (rather than relative to the ancestral *MxB* sequence). We use green font to highlight frameshifting insertions ("\") and deletions ("/"), and red font to highlight stop codons (in-frame stop codons are shown using a star symbol; stop codons relative to the ancestral *MxB* reading frame are highlighted in the nucleotide sequence). **(A)** We show a representative alignment segment from the first coding exon of human, dog, cat and tiger *MxB*, showing an 11 bp insertion (green font) in that results in a frameshift and multiple stop codons including one at the 30^th^ amino acid. Cat and tiger genomes retain sequences matching most of the *MxB* exons; over the alignable sequences, we find that cat and tiger *MxB* pseudogenes share a total of 14 frameshifting insertion/deletions and three in-frame stop codons, indicating that the felid *MxB* gene pseudogenized at least 10.8 million years ago before *F*. *catus* and *P*. *tigris* diverged at the base of the Felidae [64]. **(B)** The armadillo genome contains exons 2 and 5–9 of *MxB*, which together contain 4 stop codons and 6 frameshifting insertion/deletions. We show a representative fragment of the sixth coding exon. Although we are unable to date this pseudogenization event, the multiple stop codons suggest that this loss is not recent. **(C)** The squirrel genome has only retained *MxB* coding exon 5 and part of exon 4, with a total of 3 frameshifting mutations and one stop codon. We show a portion of exon 4.
(EPS)

S3 Fig Recurrent gene conversion of rodent *Mx* genes scrambles phylogenetic relationships and extends into intronic regions. **(A)** Maximum-likelihood derived PHYML phylogenies of rodent *MxA*-like genes based on nucleotide alignments of segments with uniform evolutionary history (evidence ratio >100) as identified by GARD analysis from selected rodent species, rabbit and squirrel: nucleotides 31–226; B. 227–407; C. 408–894 and D. 895–2148 (with poorly-aligning loop L4 removed from the alignment). Bootstrap values for nodes with support greater than 50% are shown. Supported breakpoints (***, *P* < 0.01; **, *P* < 0.05) and the location of GARD segments are shown on a linear schematic of the mouse Mx1 protein, colored as in Fig 1B. **(B)** and **(C)** We used the mVISTA tool [41] to compare genomic sequences of pairs of *Mx* paralogs from Chinese hamster and chinchilla, taking sequences starting 5kb outside of the protein-coding region for each gene. Each plot represents the genomic sequence encompassing one *Mx* paralog on the X axis, with the Y axis showing the percent identity of a pairwise alignment with the genomic sequence encompassing the other *Mx* paralog from the same species. Vertical blue shaded regions indicate protein-coding exons and vertical orange shading indicates "conserved non-coding segments (CNS)" (stretches of > = 100bp with > = 70% identity). Other colored rectangles above the plot indicate repeats. Note that high levels of similarity between paralogs extend into many *Mx* introns.
(EPS)

S4 Fig Comparison of human *MxA* and *MxB*. A pairwise comparison of the human *MxA* and *MxB* gene coding sequence is displayed as boxes, where identical (grey) or divergent (black) nucleotide sequence is indicated. The region spanning coding exon 2 shows robust homogenization. The intron/exon context of this region is highlighted. We also show a VISTA plot comparing the genomic regions containing human *MxA* and *MxB* (see legend to S3 Fig).
(EPS)

S1 Dataset Primate MxB alignment. Fasta-formatted nucleotide alignment of primate *MxB* sequences. This alignment was used for the analyses shown in Fig 1 and S1A Fig. The last 14 amino acids of MxB were not analyzed.
(FASTA)

S2 Dataset Primate MxA alignment. Fasta-formatted nucleotide alignment of primate *MxA* sequences with indel positions removed. This alignment was used for the analyses shown in S1B Fig.
(FASTA)

S3 Dataset Alignment of MxA and MxB sequences from selected mammals. Fasta-formatted amino acid alignment of selected mammalian MxA and MxB sequences used for the phylogenetic analysis shown in Fig 2B. We excluded the N-terminus and loop L4 from the alignment, because these regions do not align well between MxA and MxB.
(FASTA)

S4 Dataset Alignment of *MxA*-like sequences from selected rodent species and rabbit. Fasta-formatted nucleotide alignment of *MxA* and *MxB* sequences used for analysis shown in Fig 3 and S3 Fig. We excluded the N-terminus and loop L4 from the alignment, because these regions do not align well between rodent/rabbit *MxA*-like sequences.
(FASTA)

S5 Dataset Alignment of *MxA* and *MxB* sequences from selected primate, carnivore and ungulate species. Fasta-formatted nucleotide alignment of *MxA*-like sequences used for analysis shown in Fig 4. We excluded the N-terminus and loop L4 from the alignment, because these regions do not align well between *MxA* and *MxB*.
(FASTA)
