# Supplementary material for: Evolutionary Analyses Suggest a Function of MxB Immunity Proteins Beyond Lentivirus Restriction
Source: PLoS Pathog. 2015 Dec 10;11(12):e1005304. doi: 10.1371/journal.ppat.1005304 (PMC4687636; doi:10.1371/journal.ppat.1005304)
Supplement: S1 Table — Genbank accession numbers for all Mx gene sequences used for the analyses in this study. (DOCX) [file ppat.1005304.s001.docx]

**Table S1. Identifiers for sequences used in this study**

| Sequence ID | Species | | *Mx* | Figure |
| --- | --- | --- | --- | --- |
|  | Common name | Binomial name |  |  |
| XM_007947318.1 | aardvark | *Orycteropus afer afer* | *A* | 2 |
| XM_007947319.1 | aardvark | *Orycteropus afer afer* | *B* | 2 |
| XM_004466306.1 | armadillo | *Dasypus novemcinctus* | *A* | 2 |
| XM_010958347.1 | Bactrian camel | *Camelus bactrianus* | *A* | 3 |
| XM_010958348.1 | Bactrian camel | *Camelus bactrianus* | *B* | 3 |
| XM_006886764.1 | Cape elephant shrew | *Elephantulus edwardii* | *A* | 2 |
| XM_006886766.1 | Cape elephant shrew | *Elephantulus edwardii* | *B* | 2 |
| XM_005375852.1 | chinchilla | *Chinchilla lanigera* | *A* | S3 |
| XM_005375729.1 | chinchilla | *Chinchilla lanigera* | *A* | S3 |
| XM_007631120.1 | Chinese hamster | *Cricetulus griseus* | *A* | S3 |
| XM_007631124.1 | Chinese hamster | *Cricetulus griseus* | *A* | S3 |
| XM_006156375.1 | Chinese tree shrew | *Tupaia chinensis* | *A* | 2 |
| XM_006156376.1 | Chinese tree shrew | *Tupaia chinensis* | *B* | 2 |
| XM_004602516.1 | common shrew | *Sorex araneus* | *A* | 2 |
| XM_004602517.1 | common shrew | *Sorex araneus* | *B* | 2 |
| NM_173940.2 | cow | *Bos taurus* | *A* | 2, 3 |
| NM_173941.2 | cow | *Bos taurus* | *B* | 2, 3 |
| XM_005638735.1 | dog | *Canis lupus familiaris* | *A* | 2 |
| NM_001003133.1 | dog | *Canis lupus familiaris* | *B* | 2 |
| XM_003418975.2 | elephant | *Loxodonta africana* | *A* | 2 |
| XM_010598660.1 | elephant | *Loxodonta africana* | *A* | 2 |
| ENSLAFT00000009018 | elephant | *Loxodonta africana* | *B* | 2 |
| XM_004819178.1 | ferret | *Mustela putorius* | *A* | 3 |
| XM_004819177.1 | ferret | *Mustela putorius* | *B* | 3 |
| XM_006916729.1 | flying fox | *Pteropus alecto* | *A* | 2 |
| XM_006916730.1 | flying fox | *Pteropus alecto* | *B* | 2 |
| XM_005001022.1 | guinea pig | *Cavia porcellus* | *A* | S3 |
| XM_003463910.2 | guinea pig | *Cavia porcellus* | *A* | S3 |
| DQ218274.1 | hispid cotton rat | *Sigmodon hispidus* | *A* | S3 |
| DQ218273.1 | hispid cotton rat | *Sigmodon hispidus* | *A* | S3 |
| NM_001082492.1 | horse | *Equus caballus* | *A* | 2 |
| XM_005606159.1 | horse | *Equus caballus* | *B* | 2 |
| ENSDORG00000003337 | kangaroo rat | *Dipodomys ordii* |  | 2, 3, S3 |
| XM_007172715.1 | Minke whale | *Balaenoptera acutorostrata* | *A* | 3 |
| XM_007172717.1 | Minke whale | *Balaenoptera acutorostrata* | *B* | 3 |
| NM_010846.1 | mouse1 | *Mus musculus* | *A* | 2, 3, S3 |
| BC007127.1 | mouse2 | *Mus musculus* | *A* | 2, 3, S3 |
| XM_004842350.1 | naked mole rat | *Heterocephalus glaber* | *A* | S3 |
| XM_004842462.1 | naked mole rat | *Heterocephalus glaber* | *A* | S3 |
| XM_002925877.2 | panda | *Ailuropoda melanoleuca* | *A* | 3 |
| XM_011233153.1 | panda | *Ailuropoda melanoleuca* | *B* | 3 |
| ENSOANT00000010270 | platypus | *Ornithorhynchus anatinus* | *X* | 2 |
| ENSOANT00000010275 | platypus | *Ornithorhynchus anatinus* | *X* | 2 |
| XM_005370126.1 | prairie vole | *Microtus ochrogaster* | *A* | S3 |
| XM_005370124.1 | prairie vole | *Microtus ochrogaster* | *A* | S3 |
| XM_008252474.1 | rabbit | *Oryctolagus cuniculus* | *A* | S3 |
| XM_008254005.1 | rabbit | *Oryctolagus cuniculus* | *A* | S3 |
| ENSRNOT00000039876 | rat | *Rattus norvegicus* | *A* | 2, 3, S3 |
| ENSRNOT00000002695 | rat | *Rattus norvegicus* | *A* | 2, 3, S3 |
| ENSSTOG00000012846 | squirrel | *Ictidomys tridecemlineatus* | *A* | 2, 3, S3 |
| XM_008571220.1 | Sunda flying lemur | *Galeopterus variegatus* | *A* | 2 |
| XM_008571218.1 | Sunda flying lemur | *Galeopterus variegatus* | *B* | 2 |
| XM_004697318.1 | tenrec | *Echinops telfairi* | *A* | 2 |
| XM_004697319.1 | tenrec | *Echinops telfairi* | *B* | 2 |
| XM_004406553.1 | walrus | *Odobenus rosmarus* | *A* | 3 |
| XM_004406588.1 | walrus | *Odobenus rosmarus* | *B* | 3 |
| ENSTGUT00000005547 | zebra finch | *Taeniopygia guttata* | *X* | 2 |

X = *MxAB* from outgroup lineage
