# Supplementary material for: Evolutionary Analyses Suggest a Function of MxB Immunity Proteins Beyond Lentivirus Restriction
Source: PLoS Pathog. 2015 Dec 10;11(12):e1005304. doi: 10.1371/journal.ppat.1005304 (PMC4687636; doi:10.1371/journal.ppat.1005304)
Supplement: S2 Table — We report the source cell lines from which the MxA and/or MxB sequences were obtained for this study. In all cases, MxA and MxB gene sequences were derived from the same sample. Primate MxB sequences cloned in this study are indicated (Genbank Accession numbers KT698228-KT698252). WNPRC, Washington National Primate Research Center. TNPRC, Tulane National Primate Research Center. (DOCX) [file ppat.1005304.s002.docx]

**Table S2. Primate species and cell lines used in this study**

| *Common name* | *Binomial*  *name* | *Sample source* | *Mx* | *New seq.* | *Sequence ID* |
| --- | --- | --- | --- | --- | --- |
| crab-eating macaque | *Macaca fascicularis* | M05221 (WNPRC) | *A* | No | JX297237.1 |
|  |  |  | *B* | Yes | KT698241 |
| rhesus macaque | *Macaca mulatta* |  | *A* | No | NP_001073161 |
|  |  |  | *B* | No | NM_001079696.1 |
| Barbary macaque | *Macaca sylvanus* | GB72  (Dietrich et al., 2010) | *A* | No | JX297238.1 |
|  |  |  | *B* | Yes | KT698242 |
| sooty mangabey | *Cercocebus atys* | G077  (TNPRC) | *A* | No | JX297231.1 |
|  |  |  | *B* | Yes | KT698232 |
| African green monkey | *Chlorocebus tantalus* | CRL1651 (ATCC) | *A* | No | JX297232.1 |
|  |  |  | *B* | Yes | KT698235 |
| African green monkey | *Chlorocebus sabeus* |  | *A* | No | XM_007968734.1 |
|  |  |  | *B* | No | XM_007968671.1 |
| talapoin monkey | *Miopithecus talapoin* | PR00716 (Coriell) | *A* | No | JX297239.1 |
|  |  |  | *B* | Yes | KT698244 |
| Francois’ leaf monkey | *Trachypithecus francoisi* | PR01099 (Coriell) | *A* | No | JX297248.1 |
|  |  |  | *B* | Yes | KT698252 |
| colobus monkey | *Colobus guereza* | PR00980 (Coriell) | *A* | No | JX297233.1 |
|  |  |  | *B* | Yes | KT698236 |
| human | *Homo sapiens* |  | *A* | No | NM_001144925 |
|  |  |  | *B* | No | XM_011529572.1 |
| bonobo | *Pan paniscus* | AG05253 (Coriell) | *A* | No | JX297241.1 |
|  |  |  | *B* | No | XM_008977609.1 |
| chimpanzee | *Pan troglodytes* | AG06939 (Coriell) | *A* | No | JX297242.1 |
|  |  |  | *B* | Yes | KT698247 |
| gorilla | *Gorilla gorilla* | AG05251 (Coriell) | *A* | No | JX297234.1 |
|  |  |  | *B* | Yes | KT698238 |
| orangutan | *Pongo pygmaeus* | AG05252 (Coriell) | *A* | No | JX297244.1 |
|  |  |  | *B* | No | XM_009233982.1 |
| agile siamang | *Hylobates agilis* | PR00773 (Coriell) | *A* | No | JX297235.1 |
|  |  |  | *B* | Yes | KT698239 |
| island siamang | *Symphalangus syndactylus* | PR00722 (Coriell) | *A* | No | JX297247.1 |
|  |  |  | *B* | Yes | KT698251 |
| white-cheeked gibbon | *Nomascus leucogenys* | PR01037 (Coriell) | *A* | No | JX297240.1 |
|  |  |  | *B* | Yes | KT698245 |
| common marmoset | *Callithrix jacchus* |  | *A* | No | XM_002761438 |
|  |  |  | *B* | No | XM_008986726.1 |
| red-handed tamarin | *Saguinus midas* | PR00550 (Coriell) | *A* | No | JX297245.1 |
|  |  |  | *B* | Yes | KT698249 |
| three-striped night monkey | *Aotus trivirgatus* | CRL1556 (ATCC) | *A* | No | JX297229.1 |
|  |  |  | *B* | Yes | KT698230 |
| common squirrel monkey | *Saimiri sciureus* | AG05311 (Coriell) | *A* | No | JX297246.1 |
|  |  |  | *B* | Yes | KT698250 |
| Bolivian red howler monkey | *Alouatta sara* | PR00708 (Coriell) | *A* | No | JX297228.1 |
|  |  |  | *B* | Yes | KT698229 |
| common woolly monkey | *Lagothrix lagotricha* | AG05356 (Coriell) | *A* | No | JX297236.1 |
|  |  |  | *B* | Yes | KT698240 |
| dusky titi | *Callicebus moloch* | AG06115 (Coriell) | *A* | No | JX297230.1 |
|  |  |  | *B* | Yes | KT698231 |
| white-faced saki | *Pithecia pithecia* | PR00239 (Coriell) | *A* | No | JX297243.1 |
| mandrill | *Mandrillus sphinx* | PR00399  (Coriell) | *B* | Yes | KT698243 |
| baboon | *Papio anubis* |  | *B* | No | XM_009202223.1 |
| patas monkey | *Erythrocebus patas* | AG06116  (Coriell) | *B* | Yes | KT698237 |
| lesser white nosed monkey | *Cercopithecus petaurista* | PR00949  (Coriell) | *B* | Yes | KT698234 |
| mustached guenon | *Cercopithecus cephus* | PR00527  (Coriell) | *B* | Yes | KT698233 |
| Allen's swamp monkey | *Allenopithecus nigroviridis* | PR01231  (Coriell) | *B* | Yes | KT698228 |
| golden snub-nosed monkey | *Rhinopithecus roxellana* |  | *B* | No | XM_010354512.1 |
