# Supplementary material for: Evolutionary Analyses Suggest a Function of MxB Immunity Proteins Beyond Lentivirus Restriction
Source: PLoS Pathog. 2015 Dec 10;11(12):e1005304. doi: 10.1371/journal.ppat.1005304 (PMC4687636; doi:10.1371/journal.ppat.1005304)
Supplement: S3 Table — Please see Methods for details about how the analyses were carried out and residues putatively evolving under diversifying selection identified. (DOCX) [file ppat.1005304.s003.docx]

**Table S3. Sites with dN/dS > 1 as detected by REL (GARD segments analyzed individually)**

| **Codon** | **E[dS]** | **E[dN]** | **Normalized E[dN-dS]** | **Posterior Probability** | **Bayes Factor** |
| --- | --- | --- | --- | --- | --- |
| ***MxB* (n=32), all simian primates, GARD segment 1** | | | | | |
| 6 | 0.718175 | 1.46454 | 0.746364 | 0.994125 | 141.454 |
| 7 | 0.557419 | 1.44409 | 0.886676 | 0.988194 | 69.9732 |
| 37* | 0.731904 | 1.46764 | 0.735736 | 0.995399 | 180.839 |
| 51 | 0.662083 | 1.46872 | 0.80664 | 0.998293 | 488.917 |
| ***MxB* (n=32), all simian primates, GARD segment 2** | | | | | |
| 202 | 0.645625 | 1.72428 | 1.07865 | 0.933215 | 171.307 |
| 301 | 0.612405 | 1.76145 | 1.14904 | 0.966665 | 355.509 |
| 348 | 0.591834 | 1.70945 | 1.11762 | 0.932598 | 169.626 |
| 355 | 0.558179 | 1.67062 | 1.11244 | 0.909157 | 122.694 |
| 462 | 0.902138 | 1.62834 | 0.726206 | 0.811876 | 52.9077 |
| 499 | 0.603605 | 1.6213 | 1.0177 | 0.86317 | 77.3374 |
| 528 | 0.586817 | 1.75766 | 1.17085 | 0.969261 | 386.573 |
| 600 | 0.584856 | 1.75751 | 1.17266 | 0.969576 | 390.691 |
| 613 | 0.746092 | 1.65231 | 0.906215 | 0.859837 | 75.2067 |
| 616 | 0.760852 | 1.65552 | 0.894667 | 0.859237 | 74.834 |
| 628 | 0.579743 | 1.75646 | 1.17671 | 0.96984 | 394.219 |
| 682 | 0.630794 | 1.55098 | 0.920185 | 0.805534 | 50.7825 |
| ***MxB* (n=17), Old World monkeys GARD segment 1** | | | | | |
| 44 | 0.820312 | 7.90337 | 7.08305 | 0.998301 | 150.461 |
| ***MxB* (n=17), Old World monkeys GARD segment 2** | | | | | |
| 301 | 0.771308 | 3.50858 | 2.73727 | 0.948386 | 280.421 |
| 334 | 0.995108 | 3.47488 | 2.47978 | 0.910089 | 154.476 |
| 463 | 0.626225 | 3.5005 | 2.87427 | 0.963982 | 408.451 |
| 466 | 0.640918 | 3.47389 | 2.83297 | 0.954753 | 322.022 |
| 528 | 0.737265 | 3.56016 | 2.82289 | 0.967205 | 450.092 |
| 532 | 0.547158 | 3.4996 | 2.95244 | 0.97265 | 542.741 |
| 600 | 0.616312 | 3.51942 | 2.90311 | 0.970599 | 503.815 |
| 616 | 2.07363 | 3.51013 | 1.43651 | 0.800916 | 61.396 |
| 628 | 0.629191 | 3.56157 | 2.93238 | 0.981035 | 789.454 |
| 639 | 0.66809 | 3.55972 | 2.89163 | 0.975823 | 615.959 |
| 682 | 0.809436 | 3.56026 | 2.75082 | 0.957875 | 347.019 |
| 690 | 0.674957 | 3.49944 | 2.82448 | 0.957892 | 347.169 |
| ***MxB* (n=15), hominoids and New World monkeys GARD segment 1** | | | | | |
| 6 | 0.792221 | 1.4726 | 0.680381 | 0.992665 | 180.765 |
| 20 | 0.820138 | 1.4786 | 0.658462 | 0.994218 | 229.662 |
| 46 | 0.950907 | 1.47935 | 0.528441 | 0.996339 | 363.475 |
| 51 | 0.760722 | 1.47459 | 0.713864 | 0.995678 | 307.683 |
| ***MxB* (n=15), hominoids and New World monkeys GARD segment 2** | | | | | |
| 164 | 0.573264 | 1.31706 | 0.7438 | 0.918783 | 72.3554 |
| 355 | 0.581179 | 1.3195 | 0.738324 | 0.917415 | 71.051 |
| 499 | 0.655775 | 1.32598 | 0.670207 | 0.891441 | 52.521 |
| ***MxB* (n=7), New World monkeys GARD segment 1** | | | | | |
| no selected sites | | |  |  |  |
| ***MxB* (n=7), New World monkeys GARD segment 2** | | | | | |
| no selected sites | | |  |  |  |
| **MxB (n=25), 'matched' set of simian primates, GARD segment 1 (aa 1-94)** | | | | | |
| 37 | 0.743532 | 1.76771 | 1.02418 | 0.981627 | 83.2105 |
| 46 | 1.08139 | 1.79509 | 0.713703 | 0.98826 | 131.103 |
| 51 | 0.688898 | 1.74407 | 1.05517 | 0.972193 | 54.45 |
| **MxB (n=25), 'matched' set of simian primates, GARD segment 2 (aa 95-715)** | | | | | |
| 164 | 0.863631 | 1.34209 | 0.478461 | 0.918519 | 66.2462 |
| 175 | 0.660396 | 1.33495 | 0.674558 | 0.957508 | 132.423 |
| 196 | 0.613042 | 1.33369 | 0.720643 | 0.966841 | 171.35 |
| 202 | 0.679035 | 1.35486 | 0.675827 | 0.969629 | 187.615 |
| 301 | 0.634663 | 1.35608 | 0.721414 | 0.98053 | 295.958 |
| 348 | 0.622615 | 1.35493 | 0.732317 | 0.982288 | 325.904 |
| 355 | 0.575243 | 1.3539 | 0.778656 | 0.99196 | 725.022 |
| 462 | 0.902655 | 1.35295 | 0.450299 | 0.918282 | 66.0365 |
| 499 | 0.622364 | 1.35262 | 0.730254 | 0.980396 | 293.884 |
| 528 | 0.60765 | 1.3543 | 0.746652 | 0.985076 | 387.888 |
| 532 | 0.568758 | 1.33456 | 0.765806 | 0.977273 | 252.697 |
| 587 | 0.558842 | 1.33165 | 0.77281 | 0.977011 | 249.756 |
| 600 | 0.611427 | 1.356 | 0.744578 | 0.985656 | 403.813 |
| 602 | 0.644829 | 1.33395 | 0.689116 | 0.960094 | 141.383 |
| 609 | 0.734509 | 1.33365 | 0.599145 | 0.940214 | 92.4181 |
| 613 | 0.785025 | 1.35336 | 0.568339 | 0.944797 | 100.579 |
| 616 | 0.575357 | 1.33253 | 0.757178 | 0.974136 | 221.339 |
| 628 | 0.598975 | 1.33911 | 0.740139 | 0.974416 | 223.826 |
| 639 | 0.572738 | 1.33817 | 0.765434 | 0.979408 | 279.509 |
| **MxB (n=25), 'matched' set of simian primates, N-terminus (aa 1-83)** | | | | | |
| 6 | 0.796606 | 1.88761 | 1.091 | 0.932217 | 50.4843 |
| 37 | 0.797465 | 1.9321 | 1.13463 | 0.961514 | 91.7093 |
| 44 | 0.906025 | 1.91761 | 1.01158 | 0.935751 | 53.4628 |
| 46 | 0.845585 | 1.97957 | 1.13399 | 0.986799 | 274.408 |
| 51 | 0.789943 | 1.88751 | 1.09756 | 0.933083 | 51.1848 |
| **MxB (n=25), 'matched' set of simian primates, Loop L4 (aa 580-621)** | | | | | |
| 600 | 0.684243 | 1.33097 | 0.646725 | 0.982056 | 119.726 |
| **MxA (n=25), 'matched' set of simian primates, GARD segment 1 (aa 1-253)** | | | | | |
| 3 | 0.491227 | 1.59471 | 1.10348 | 0.998178 | 5010.51 |
| 20 | 0.732044 | 1.40869 | 0.676641 | 0.846701 | 50.5193 |
| 34 | 0.545706 | 1.59278 | 1.04707 | 0.989543 | 865.577 |
| 42 | 0.59035 | 1.55823 | 0.96788 | 0.958252 | 209.95 |
| 131 | 0.500683 | 1.55573 | 1.05505 | 0.970436 | 300.242 |
| 135 | 0.506606 | 1.38706 | 0.880456 | 0.855424 | 54.1193 |
| 141 | 0.738178 | 1.57026 | 0.832078 | 0.937373 | 136.904 |
| 196 | 0.50043 | 1.38063 | 0.880195 | 0.851659 | 52.5137 |
| **MxA (n=25), 'matched' set of simian primates, GARD segment 2 (aa 254-662)** | | | | | |
| 301 | 0.587693 | 2.28308 | 1.69539 | 0.968525 | 76.3392 |
| 416 | 0.545449 | 2.91046 | 2.36501 | 0.991895 | 303.602 |
| 540 | 0.808463 | 2.79731 | 1.98884 | 0.963246 | 65.0176 |
| 566 | 0.606359 | 3.12925 | 2.52289 | 0.995346 | 530.57 |
| **MxA (n=25), 'matched' set of simian primates, N terminus (aa 1-44)** | | | | | |
| 3 | 0.736751 | 4.64857 | 3.91182 | 0.996328 | 756.355 |
| 35 | 1.18219 | 4.58087 | 3.39868 | 0.960322 | 67.4646 |
| **MxA (n=25), 'matched' set of simian primates, Loop L4 (aa 534-572)** | | | | | |
| no selected sites | |  |  |  |  |

Note that although residue 37 of MxB (asterisked) is highlighted by the all primate REL analysis, it is not identified in the all primate PAML analysis. Moreover, it is not highlighted in any Old World Monkey analysis, the clade in which we would expect it to be identified if the signal was due to an ‘arms-race’ with primate lentiviruses.
